# Phytochemical analysis and biological investigation of *Cheilanthes tenuifolia* (Burm.f.) Swartz

**DOI:** 10.3389/fphar.2024.1366889

**Published:** 2024-04-04

**Authors:** Umme Habiba Juhi, Heba A. S. El-Nashar, Abdullah Al Faruq, Md. Shimul Bhuia, Irin Sultana, Syedul Alam, Farah Abuyousef, Na’il Saleh, Mohamed El-Shazly, Muhammad Torequl Islam

**Affiliations:** ^1^ Department of Pharmacy, Southern University Bangladesh, Chattogram, Bangladesh; ^2^ Department of Pharmacognosy, Faculty of Pharmacy, Ain Shams University, Cairo, Egypt; ^3^ Department of Pharmacy, Bangabandhu Sheikh Mujibur Rahman Science and Technology University, Gopalganj, Bangladesh; ^4^ Bioluster Research Center, Dhaka, Bangladesh; ^5^ Forest Botany Division, Bangladesh Forest Research Institute (BFRI), Chattogram, Bangladesh; ^6^ Department of Chemistry, College of Science, United Arab Emirates University, Al Ain, United Arab Emirates; ^7^ Pharmacy Discipline, Khulna University, Khulna, Bangladesh

**Keywords:** anti-inflammatory, *Cheilanthes tenuifolia*, secondary metabolites, antimicrobial, membrane stabilization, anti-atherothrombotic, radical scavenging

## Abstract

**Introduction:** *Cheilanthes tenuifolia* is an evergreen ornamental small fern, belonging to the family Pteridaceae, that grows in warm and rocky regions worldwide. Many species of *Cheilanthes* genus are evidently endowed with important phytochemicals and bioactivities. This study aimed to perform a preliminary phytochemical analysis of *Cheilanthes tenuifolia* leaves alongside an evaluation of free radical scavenging, anti-inflammatory, antimicrobial, and clot lysis activities of extract fractions.

**Materials and methods:** A preliminary phytochemical analysis was done after fractionation of ethanolic extract (ECT) with *n*-hexane (HCT) and chloroform (CCT). Then, 2,2-diphenyl-1-picrylhydrazyl (DPPH) radical scavenging, egg albumin and RBC membrane stabilization tests, disc diffusion, and human blood clot lysis assays were performed.

**Results:** Phytochemical investigations suggested that the plant is rich in alkaloids, glycosides, tannins, and flavonoids. All obtained fractions exhibited concentration-dependent radical scavenging, inhibition of egg protein denaturation and RBC membrane lysis capacities. Except for antifungal tests, ECT exhibited better DPPH radical scavenging, anti-inflammatory, antibacterial, and clot lysis capacities than HCT and CCT fractions. However, all fractions exhibited a mild anti-inflammatory activity.

**Conclusion:** *C. tenuifolia* might be a good source of antioxidant, anti-microbial, and anti-atherothrombotic agents. Further studies are required to isolate and characterize the active principles liable for each bioactivity, along with possible molecular interactions.

## 1 Introduction

Recently, the herbal remedies have been recognized as alternative medical treatments for managing primary healthcare among 80% of the world’s populations, especially in low- and medium-income countries (El-Nashar et al., 2021; El-Shawi et al., 2023). It is due to the fact that most often synthetic drugs result in many unavoidable adverse events, grow resistance (e.g., antibiotics) and tolerance (e.g., narcotic drugs) due to repeated uses, and thereby reduce safety and efficacy (Ishaq et al., 2022; El-Nashar et al., 2023b; Younis et al., 2023).

The use of pteridophytes (e.g., aka, ferns and fern allies) is ancient (>2000 years) due to their many beneficial properties. Pteridaceae is recognized as an important family among the pteridophytes. Many species in this family are rich with alkaloids, glycosides, and flavonoids and have promising medicinal features, such as antioxidant, anti-cancer, anti-inflammatory, antimicrobial, antidiabetic, and neurobiological effects (Bhuia et al., 2023a). Cheilanthes tenuifolia (Burm.f.) Swartz, which belongs to the family Pteridaceae, is a petite, evergreen fern that has the ability to reach a height of 70 cm. The delicate lip fern is a species of fern loacal to North America that is considered an ornamental plant (Mahfuz et al., 2019). It grows properly in open, warm, moist, shady, rocky regions and is often found in small crevices high up on cliffs (Sen and Mukhopadhyay, 2014; Patel and Reddy, 2018). It is found in many regions of the world, including Australia, China, Malaysia, Bangladesh, Nepal, Cambodia, Laos, New Zealand, the Philippines, Polynesia, Sri Lanka, Taiwan, Uruguay, Thailand, Vietnam, Tasmania, and India. September to November is considered the growing season for the plants. In India, there are over 30 species of this plant. The fronds are 63 cm long and 17 cm wide, with a dark red-brown stipe and rachis that are smooth or have sparse hairs consisting of 2–13 cells and very few slender scales. The lamina is pentagonal, triangular, or ovate, with 3-4 pinnates at the base and 3 pinnates for most of its length. The larger pinnae are triangular-ovate, the pinnules are lanceolate or ovate, and the ultimate pinnules may have a slightly caudate shape. The margins are either lobed or entire. The upper and lower surfaces of the lamina have very few, short (less than 0.5 mm), pointed hairs consisting of 2 or 3 cells, and are occasionally almost hairless. The spores are tetrahedral or rounded-tetrahedral, granulose, and trilete, with a varying degree of reticulate-echinate ornamentation, and have a diameter of 38–53 µm with 32 per sporangium.

Ancient people (prehistoric times) used the rhizome juice of ferns for gastrointestinal disorders (including peptic ulcer), cuts, and wounds. Traditionally, C. tenuifolia leaf juice is mixed with hot water and honey to cure throat pain (Augustin and Thomas, 2015). Its leaf and stem decoctions are used for healthy hair (Hanum and Hamzah, 1999). The tribes of Northeast India use its rhizomes and root extracts as general tonics (Benniamin, 2011; Singh and Upadhyay, 2014). One study reports that the plant contains important phytochemical groups, like steroids, alkaloids, flavonoids, triterpenoids, phenolic compounds, and tannins (Ghorpade et al., 2015). To date, two important flavonoids such as quercetin and rutin have been extracted from an ethyl acetate-soluble extract of C. tenuifolia (Jarial et al., 2018). The plant-derived compound quercetin is a natural aglycone of rutin. It is frequently used in dietary supplements due to its many important and promising bioactivities, including antioxidant, anti-inflammatory, immunomodulatory, antimicrobial, anti-cancer, antidiabetic, antiallergy, antihypertensive, and organ-protective (e.g., brain, heart, liver, kidney, and GIT tract) effects (Ahmed et al., 2022). A recent investigation proposes that methanolic whole plant extract possesses a significant amount of phenolics and flavonoids and has demonstrated strong antioxidant, cytotoxic, membrane-stabilizing, and thrombolytic activity (Mahfuz et al., 2019). This study also confirmed that the plant contains stigmasterol. Therefore, there is a lack of adequate scientific studies on this hopeful medicinal plant and there is no detailed information on isolated phytochemicals, anti-inflammatory activity and its underlying mechanisms, antifungal properties, antibacterial effects against huge number of pathogenic bacteria and the molecular mechanisms of each activity.

Knowing the overall facts, this study aimed to do a preliminary phytochemical analysis along with the assessment of the radical scavenging, anti-inflammatory, antimicrobial, and clot lysis capacities of Cheilanthes tenuifolia leaf extracts and this study also evaluated the rectitude of the previous findings of the therapeutic activity of the plant.

## 2 Experimental

### 2.1 Collection, identification, and extraction of plant materials

Fresh leaves were gathered from the Bayazid hill tracts in Chittagong during July and August, which is the period when the plant grows the most. Before mass collection, the plant is identified by a taxonomist at the Bangladesh Forest Research Institute Herbarium (BFRIH), Chittagong [Voucher No. BFRIH-SA (577)]. The decayed leaves, stems, dust, and other parts of the plant were removed with great care. Next, the plant components were rinsed using a continuous flow of tap water and dried in the shade at a temperature lower than 40°C. After drying, the materials were crushed into a rough powder and placed in an airtight container that is amber in color. This container was then kept in a cool and dry place until the extraction process began.

A total of 200 g of leaf powder was extracted with 1,000 mL of absolute ethanol at solvent ratio of 1:5 using a Soxhlet extractor a temperature of 70°C for 8 h. Then the solvent was dried using a rotary evaporator under diminished pressure. Finally, a gummy ethanolic leaf extract (ECT) was collated. The percentage yield value was determined as follows:

$$\%\text{Yield}=\left[\text{Weight of crude extract }\left(\text{gm}\right)\;/\text{ Weight of powder taken }\left(\text{gm}\right)\right]\times 100$$

### 2.2 Fractionation of crude ethanolic extract

**FIGURE 1:**
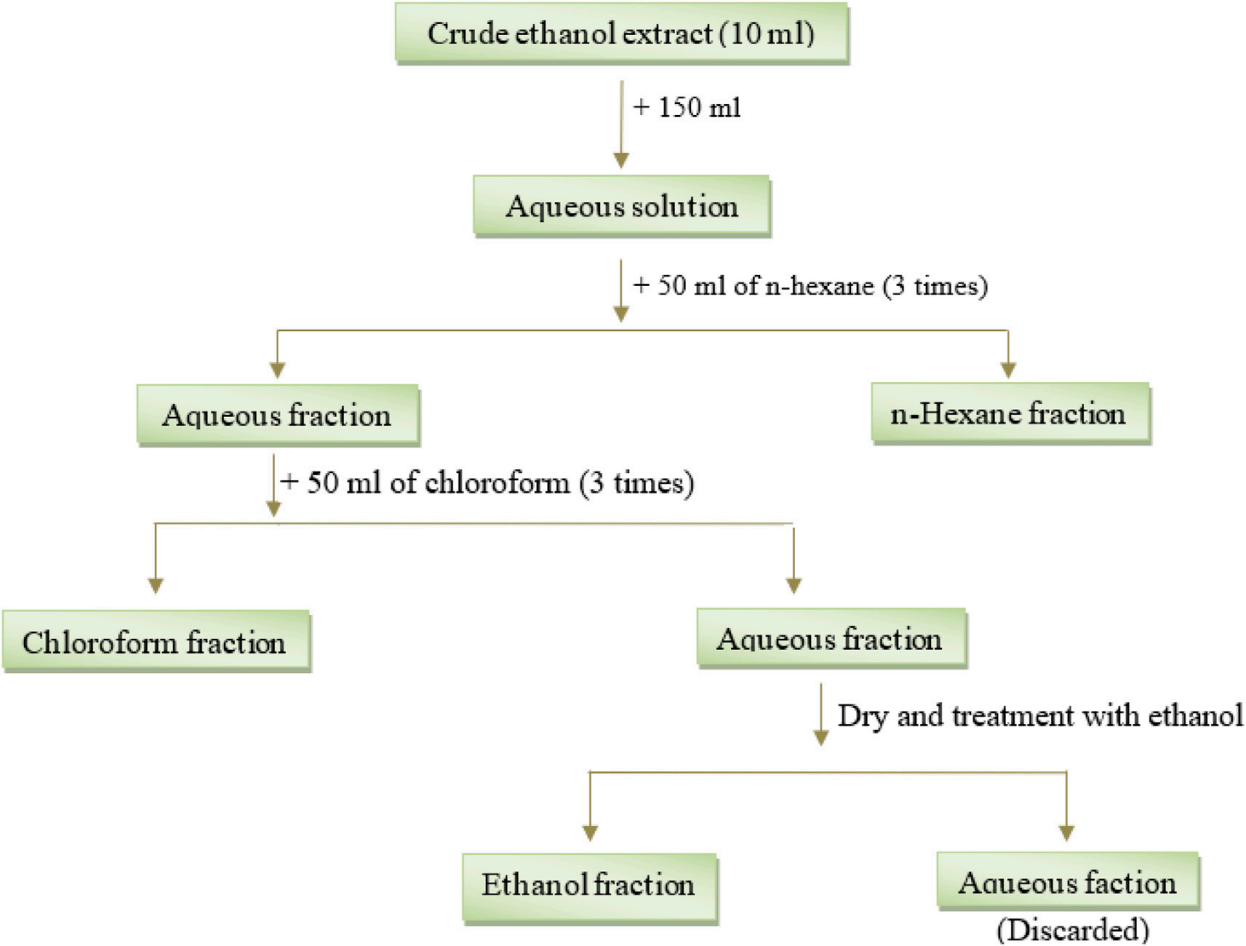
Schematic presentation of fractionation of *Cheilanthes tenuifolia* ethanolic leaf extract.

To perform solvent-solvent partitioning of ECT, 10 mL of ECT was dissolved in double-distilled water (DDW), and then fractionated with the aid of a fractionating column with n-hexane (HCT) and subsequently with chloroform (CCT). The fractionation process involved utilizing 50 mL of each solvent, for a total of 150 mL of n-hexane and chloroform. After vigorous shaking of the mixture, each fraction was allowed to stand, and the solvent layers were then separated and decanted. The remaining extract was considered a fraction of ethanol. The extracts obtained were collected, filtered, and the solvent was evaporated below 50°C temperature. As a result of the evaporation process, a sticky concentrate was obtained. The gummy concentrates were weighed and placed in an appropriately labeled and cleaned airtight container and stored at 4°C. Percentage yield values for each fraction were determined according to the above-mentioned equation. A general scheme for fractionation has been shown in Figure 1.

### 2.3 Reagents and chemicals

Streptokinase (Altepase^®^) was purchased from Beacon Pharmaceuticals Ltd., Bangladesh, while ethanol, chloroform, n-hexane, tween 80, acetyl salicylic acid, ascorbic acid, DPPH, nutrient culture media, and other necessary reagents and chemicals were purchased from Merck India.

### 2.4 Experimental animals

The study utilized young male Swiss albino mice, which were procured from the animal research branch of the Bangladesh Council of Scientific and Industrial Research (BCSIR) in Chattogram, Bangladesh. These mice had an average weight of 24–30 g and were maintained in a laboratory setting under standard conditions: a 12-h light/dark cycle, a room temperature of 25°C ± 2°C, and a relative humidity of 55%–60%. They were provided with a standard diet and had access to water *ad libitum*. To ensure their suitability for the study, the mice were acclimatized to the laboratory environment for 7 days and fasted overnight for 12 h before the experiments. All ethical considerations were taken into account, and the experimental animals were treated in accordance with the Swiss Academy of Medical Sciences and the Swiss Academy of Sciences Ethical Principles and Guidelines for Scientific Experiments with Animals (1995). Additionally, the Institutional Ethics Committee (SUB/IAEC/12.01) approved all experimental protocols.

### 2.5 Acute toxicity analysis and test concentration determination

The test dose for this study of crude extracts was selected by the acute toxicity study following the OECD guidelines using Swiss albino mice. Briefly, the crude ECT was given at doses of 500, 1,000, 2,000, and 3,000 mg/kg orally. The animals were then frequently observed for behavioral changes, toxicological symptoms, and death for 2 days (Thangjam et al., 2020).

### 2.6 Phytochemical analysis

The phytochemical screening was done according to the method described by (Batool et al., 2020).

### 2.7 Radical scavenging assay

The study assessed the ability of the extracts to scavenge free radicals using the 2,2-diphenyl-1-picrylhydrazyl (DPPH) scavenging assay, as stated by Chowdhury et al. (2010), with minor modifications. To prepare the DPPH solution, 0.004% w/v DPPH was solubilized in ethanol, and its absorbance was immediately measured. Next, 1 mL of the extract solution at a number of concentrations (20, 40, 60, 80, and 100 μg/mL) was added to 2 mL of the DPPH solution, mixed thoroughly, and allowed to stand in the dark for 30 min to complete the reaction. The same concentrations of ascorbic acid (AA) were utilized as a reference radical scavenger, while ethanol served as a blank. After the reaction time, the absorbance was measured at 517 nm using a UV spectrophotometer, and the formula given below was utilized to determine the percentage of inhibition:

$$\%\text{ radical scavenge}=\left[\text{Absorbance}\_\left(\text{Before}\right)\;–\text{ Absorbance}\_\left(\text{After}\right)\;)÷\text{Absorbance}\_\left(\text{Before}\right)\;\right]\times 100$$

The half-minimal inhibitory concentration (IC_50_) was measured utilizing non-linear regression analysis with the aid of Graph Pad Prism software.

### 2.8 Anti-inflammatory assay

#### 2.8.1 Egg albumin test

This test was performed by checking inhibitory effects on egg albumin using the method stated by (Dharmadeva et al., 2018). Briefly, 0.2 mL of egg albumin (from a fresh hen’s egg) was mixed with 2.8 mL of isosaline (0.9% NaCl, pH 6.4) and 2 mL of the test sample or standard drug. Distilled water and acetyl salicylic acid (ASA) were served as control and positive controls, respectively. The sample and standard were tested at 125, 250, and 500 μg/mL. The reaction mixtures were incubated at a temperature of 37°C ± 2°C for 15 min, after which they were heated in a water bath at a temperature of 70°C for 5 min. Following this, the mixtures were allowed to cool and were filtered through Whatmann filter paper no. 1. Using a colorimeter, the absorbance of every sample was gauged at a 660 nm wavelength. The percentage of protein denaturation inhibition was computed utilizing the following equation:

$$\%\text{inhibition of egg protein}=100\times \left(\left[\text{Vt}/\text{Vc}\right]-1\right)$$

where, Vt and Vc stand for absorbance of test sample and control, respectively. The IC_50_ values were determined as mentioned above.

#### 2.8.2 HRBC membrane stabilization test

This study was conducted using the model developed by (Shinde et al., 1999) with some minor adjustments. Initially, 5 mL of fresh blood was gathered from a healthy donor and mixed with di-potassium salt of EDTA (2.2 mg/mL). The blood cells were then accumulated by centrifugation and washed thrice with an isotonic solution (154 mM NaCl) in 10 mM sodium phosphate buffer (pH 7.4). The resulting cell suspension was re-centrifuged at 3,000 *g* for 10 min and finally re-suspended in an equal volume of isotonic buffer solution. Next, 0.5 mL of the cell suspension was added to a mixture of 5 mL of hypotonic solution (50 mM NaCl) and 0.5 mL of test or standard solution (125, 250, and 500 μg/mL) in 10 mM sodium phosphate buffered saline (pH 7.4), as specified. The control tube contained only 0.5 mL of cell suspension and 5 mL of hypotonic solution in the above-mentioned buffer. The reaction mixture was incubated for 10 min at room temperature and centrifuged at 3,000 *g* for 10 min. Finally, the optical density (OD) of the supernatant was quantified at 540 nm using a UV-visible spectrophotometer. The percentage inhibition of hemolysis was calculated using the following equation:

$$\%\text{ inhibition of hemolysis}=\left\{\left({\text{OD}}_{\text{control}}‐{\text{OD}}_{\text{test}\;\text{samples}}\right)/{\text{OD}}_{\text{control}}\right\}\times 100$$

The IC_50_ values were determined as mentioned above.

### 2.9 Anti-microbial assay

This test was done according to the model stated by (Mbaveng et al., 2008). The investigation samples were made by solubilizing the extracts of the samples in ethanol (ECT), chloroform (CCT), and n-hexane (HCT). All the samples were tested at 500 μg/disc. Ciprofloxacin (CFN) and fluconazole (FCZ) were taken as reference drugs for anti-bacterial and anti-fungal tests, respectively, at 30 μg/disc. For this study, we used 4 G (+) and 7 G (−) bacteria and 7 fungi (Table 1). For the anti-bacterial assay, we used nutrient agar media, while for the anti-fungal assay, we used potato dextrose agar media. After the inoculation of the test pathogen and after allowing the plates to solidify, respective paper discs containing the test sample or standard drug were subsequently impregnated centrally into the agar gel separately with the aid of sterile forceps to achieve complete contact with the previously cultured medium surface. Finally, all the plates were then incubated at 37°C for 24 h for the anti-bacterial test and at 25°C for 72 h for the anti-fungal test.

**TABLE 1:** List of tested pathogens.

Bacteria		Fungi
Gram (+ve) species	Gram (−ve) species	
*Bacillus cereus*	*Escherichia coli*	*Aspergillus niger*
*Bacillus megaterium*	*Pseudomonas aeruginosa*	*Blastomyces dermatitidis*
*Bacillus subtilis*	*Salmonella paratyphi*	*Pityrosporum ovale*
*Staphylococcus aureus*	*Salmonella typhi*	*Trichophyton* sp
	*Shigella dysentariae*	*Candida albicans*
	*Shigeela sonnei*	*Microsporum* sp
	*Vibrio cholera*	*Cryptococcus neoformans*

### 2.10 Clot lysis assay

This *in vitro* study was done according to the model developed by (Prasad et al., 2006). In this case, we distributed 0.5 mL of fresh blood in pre-weighed microcentrifuge tubes from the non-contraceptive or anti-coagulant receiving humans. After incubating the blood sample at 37°C for 45 min, the serum was cautiously excluded without disquieting the clot, and tubes were weighed. 100 μL of extract at 500 μg was added in each tube. 100 μL of streptokinase (equiv. 30,000 IU) and 100 μL of DW were added to the positive control and control marked tubes, respectively. After incubation of the tubes at 37°C for 90 min, the discharged fluid from each tube was carefully removed, and the tubes were reweighed. The percentage of clot lysis was calculated as follows:

$$\%\text{ Thrombolysis}=\left(\text{weight of clot after treatment }/\text{ weight of clot before treatment}\right)\times 100$$

### 2.11 Statistical analysis

Values are expressed as mean ± standard error of mean (SEM). One-way analysis of variance (ANOVA) was followed by Newman Keuls *post host* t-students test using the Graph Pad Prism software (version 6.5) considering *p* < 0.05 at 95% confidence of intervals.

## 3 Results

### 3.1 Extraction and phytochemical profile

The percentage yield of crude ECT was 5%; that of fractionated ECT, CCT, and HCT was 25, 35, and 25%, respectively. Table 2 suggests that ECT possesses alkaloids, tannins, glycosides, flavonoids, and saponins, while CCT contains alkaloids, glycosides, steroids, tannins, and flavonoids. HCT contains alkaloids, tannins, glycosides, flavonoids, saponins, and reducing sugars. All the extracts contain alkaloids, glycosides, tannins, and flavonoids. None of these extracts contain gums or amides.

**TABLE 2:** Phytochemical groups observed in different fractions of *Cheilanthes tenuifolia* leaf extract.

Fractions	Alkaloids	Glycosides	Steroids	Tannins	Flavonoids	Saponins	Reducing sugar	Gums	Amides
ECT	+++	++	‒	++	+	+	‒	‒	‒
CCT	++	++	+	++	+	‒	‒	‒	‒
HCT	++++	+	‒	++	+	+	+	‒	‒

+ = Present; ‒ = Absent; Multiple (+) sign indicates the number of test which showed presence of phytochemical group; ECT, ethanolic fraction of *cheilanthes tenuifolia*; CCT, chloroform fraction of *Cheilanthes tenuifolia*; HCT, n-hexane fraction of *Cheilanthes tenuifolia*.

### 3.2 LD_50_ study and determination of test concentration

The crude ECT up to a 3,000 mg/kg oral dose did not cause behavioral changes or toxicological symptoms, or even death, in Swiss mice. Therefore, we used the maximum test concentration of 500 μg/mL (equivalent to 500 mg/kg) as a test concentration for antimicrobial and clot lysis studies. For radical scavenging, we used the highest concentration, 100 μg/mL, while in the anti-inflammatory study, we used 500 μg/mL as a high concentration and 125 as a low concentration.

### 3.3 Radical scavenging capacity

The control showed negligible DPPH radical scavenging capacity (1.54% ± 0.11%). All the extracts demonstrated a concentration-dependent radical scavenging capacity in comparison to the control group. The highest inhibition (IC_50_ = 22.17 ± 1.90 μg/mL) was seen with ECT at all concentrations compared to CCT and HCT. The radical scavenging capacity of all the extracts was significant (*p* < 0.05) as compared to the control group. However, the reference drug AA revealed significant (*p* < 0.05), strong, and better inhibition at 20–100 μg/mL than all the test extracts (Table 3). The IC_50_ values calculated for the CCT, HCT, and AA are 39.79 ± 1.02, 78.13 ± 2.08, and 18.77 ± 1.03 μg/mL, respectively. The CI and r^2^ value of all the treatment groups are demonstrated in Table 3.

**TABLE 3:** Percentage of scavenging activity of DPPH radicals by the tested extract and controls.

Concentration (μg/mL)	Percentage inhibition of DPPH radical			
	ECT	CCT	HCT	AA
20	48.47 ± 1.78*	46.04 ± 1.31*	33.50 ± 1.93*	51.28 ± 1.11*
40	62.43 ± 1.58*	50.71 ± 1.23*	41.50 ± 0.96*	63.49 ± 1.13*
60	68.36 ± 0.93*	54.94 ± 1.07*	47.20 ± 0.57*	77.05 ± 1.67*
80	81.16 ± 0.13*	62.32 ± 1.05*	53.90 ± 1.11*	88.03 ± 1.02*
100	88.36 ± 1.23*	74.38 ± 1.01*	61.10 ± 1.09*	91.21 ± 1.08*
IC_50_ (μg/mL)	22.17 ± 1.90	39.79 ± 1.02	78.13 ± 2.08	18.77 ± 1.03
CI (μg/mL)	15.07–27.21	35.37–47.13	71.27–93.15	13.75–25.31
r^2^	0.96	0.95	0.87	0.95
Control	1.54 ± 0.11			

Values are mean ± SEM (*n* = 3); One-way ANOVA, followed by *t*-student *post hoc* test; *p < 0.05 when compared to the control (vehicle) group; ECT, ethanolic fraction of *Cheilanthes tenuifolia*; CCT, chloroform fraction of *Cheilanthes tenuifolia*; HCT, n-hexane fraction of *Cheilanthes tenuifolia*; AA, ascorbic acid.

### 3.4 Anti-inflammatory activity

#### 3.4.1 Egg albumin test

The control showed a negligible egg protein denaturation inhibitory effect (1.23% ± 0.01%). All the extracts showed concentration-dependent protein denaturation inhibitory effects in comparison to the control group. The highest inhibition (49.84% ± 0.01%) was seen by ECT at 500 μg/mL. However, the standard drug ASA exhibited significant (*p* < 0.05), strong, and better inhibition at 125–500 μg/mL than all the test extracts (Table 4). The IC_50_ values calculated for the ECT, CCT, HCT, and ASA are 511.10 ± 2.93, 1,001.07 ± 3.09, 993.03 ± 3.57, and 122.71 ± 2.09 μg/mL, respectively.

**TABLE 4:** Percentage inhibition of egg protein denaturation by the tested extract and controls.

Parameters (μg/mL)	Percentage inhibition of protein denaturation			
	ECT	CCT	HCT	ASA
125	30.52 ± 0.01*	13.62 ± 0.01*	17.32 ± 0.01*	51.73 ± 0.01*
250	41.39 ± 0.01*	18.78 ± 0.01*	20.26 ± 0.01*	60.13 ± 0.01*
500	49.84 ± 0.01*	26.13 ± 0.01*	28.10 ± 0.01*	71.52 ± 0.01*
IC_50_ (μg/mL)	511.10 ± 2.93	1,001.07 ± 3.09	993.03 ± 3.57	122.71 ± 2.09
CI (μg/mL)	477.17–527.57	945.31–1,047.73	951.19–1,023.01	113.09–147.18
r^2^	0.87	0.88	0.89	0.91
Control	1.23 ± 0.01			

Values are mean ± SEM (n = 3); One-way ANOVA, followed by *t*-student *post hoc* test; *p < 0.05 when compared to the control (vehicle) group; ECT: ethanolic fraction of *Cheilanthes tenuifolia*; CCT, chloroform fraction of *Cheilanthes tenuifolia*; HCT, n-hexane fraction of *Cheilanthes tenuifolia*; ASA, acetyl salicylic acid.

#### 3.4.2 HRBC membrane stabilization test

The control showed negligible membrane lysing inhibitory effect (1.73% ± 0.01%) on HRBCs. All the extracts showed concentration-dependent membrane lysis inhibitory effects in comparison to the control group. The highest inhibition (45.85% ± 0.01%) was seen by ECT at 500 μg/mL. However, the standard drug ASA exhibited significant (*p* < 0.05), strong, and better inhibition at 250 and 500 μg/mL than all the test extracts (Table 5). The IC_50_ values calculated for the ECT, CCT, HCT, and ASA are 587.19 ± 1.33, 2021.03 ± 3.79, 1,524.09 ± 2.96, and 209.79 ± 2.13 μg/mL, respectively.

**TABLE 5:** Percentage inhibition of membrane lysis by the tested extract and controls.

Parameters (μg/mL)	Percentage inhibition of RBC membrane lysis			
	ECT	CCT	HCT	ASA
125	11.33 ± 0.01*	3.58 ± 0.01*	3.92 ± 0.01*	43.42 ± 0.01*
250	23.51 ± 0.01*	7.56 ± 0.01*	10.92 ± 0.01*	58.61 ± 0.01*
500	45.85 ± 0.01*	11.61 ± 0.01*	21.14 ± 0.01*	70.23 ± 0.01*
IC_50_ (μg/mL)	587.19 ± 1.33	2021.03 ± 3.79	1,524.09 ± 2.96	209.79 ± 2.13
CI (μg/mL)	567.13–621.07	1843.33–2,103.03	1,453.11–1,603.10	193.39–217.13
r^2^	0.90	0.86	0.87	0.89
Control	1.73 ± 0.01			

Values are mean ± SEM (*n* = 3); One-way ANOVA, followed by *t*-student *post hoc* test; *p < 0.05 when compared to the control (vehicle) group; ECT, ethanolic fraction of *Cheilanthes tenuifolia*; CCT, chloroform fraction of *Cheilanthes tenuifolia*; HCT, n-hexane fraction of *Cheilanthes tenuifolia*; ASA, acetyl salicylic acid.

### 3.5 Anti-bacterial sensitivity

Table 6 suggests that ECT (ZI range: 8.70 ± 1.00 to 15.00 ± 1.00 mm), CCT (ZI range: 8.30 ± 0.60 to 15.30 ± 1.20 mm), and HCT (ZI range: 8.30 ± 1.50 to 12.70 ± 0.60 mm) showed sensitivity towards all the test bacteria except *V. cholerae* at 500 µg/disc. However, the extracts were more active against the Gram (+) species. The standard drug CFN inhibited the growth of all the investigated bacteria within the range of ZI 11.30 ± 1.00 to 16.00 ± 1.00 mm at 30 µg/disc.

**TABLE 6:** Zone of inhibition observed against the test bacteria in the test and standard group.

Bacteria	Zone of inhibition (mm)			
	ECT	CCT	HCT	CFN
Gram positive species				
*B. cereus*	15.00 ± 1.00	15.30 ± 1.20	9.00 ± 2.00	16.00 ± 1.00
*B. megateriuum*	11.00 ± 1.00	12.50 ± 1.80	9.70 ± 1.50	14.70 ± 1.50
*B. subtilis*	12.00 ± 1.00	13.00 ± 1.00	8.30 ± 1.50	16.00 ± 1.00
*S. aureus*	13.00 ± 0.00	13.30 ± 0.60	12.70 ± 0.60	16.70 ± 1.50
Gram negative species				
*E. coli*	8.70 ± 1.20	8.30 ± 0.60	9.30 ± 2.30	15.50 ± 0.50
*Sh. dysenteriae*	8.70 ± 1.00	8.50 ± 0.50	8.70 ± 1.20	14.70 ± 1.00
*Sh. sonnei*	10.00 ± 1.00	9.30 ± 0.58	Ni	13.80 ± 0.30
*Sal. paratyphi*	11.00 ± 1.00	Ni	Ni	12.50 ± 1.50
*Sal. typhi*	8.70 ± 0.60	Ni	Ni	13.00 ± 0.50
*P. aeruginosa*	12.00 ± 1.00	10.00 ± 1.00	12.00 ± 1.00	11.30 ± 1.00
*V. cholerae*	Ni	Ni	Ni	13.80 ± 0.30

Values are mean ± SEM (*n* = 3); ZI, Zone of inhibition (mm); ZI, below 8 mm were considered as less sensitive and were discarded; Ni, No inhibition.

### 3.6 Anti-fungal sensitivity

Table 7 suggests that ECT (ZI range: 9.00 ± 0.20 to 11.00 ± 0.50 mm), CCT (ZI range: 8.70 ± 0.20 to 12.70 ± 0.50 mm), and HCT (ZI range: 10.00 ± 0.50 to 11.70 ± 0.20 mm) showed sensitivity towards *A. niger*, *B. dermatitidis*, *C. albicans* and *P. ovale*. However, all the extracts remain insensitive at 500 µg/disc against the other test fungi. The standard drug FCZ inhibited the growth of all the test fungi within the range of ZI 11.00 ± 0.40 to 15.30 ± 0.50 mm at 30 µg/disc.

**TABLE 7:** Zone of inhibition observed against the test fungi in the test and standard group.

Fungi	Zone of inhibition (mm)			
	ECT	CCT	HCT	FCZ
*A. niger*	9.00 ± 0.20	10.00 ± 0.30	10.30 ± 0.20	14.30 ± 0.30
*B. dermatitidis*	11.00 ± 0.50	11.70 ± 0.20	11.00 ± 0.30	12.30 ± 0.30
*C. albicans*	10.00 ± 0.42	12.70 ± 0.50	11.70 ± 0.20	15.30 ± 0.50
*P. ovale*	9.00 ± 0.23	8.70 ± 0.20	10.00 ± 0.50	13.30 ± 0.30
*Tricho.* sp	Ni	Ni	Ni	11.00 ± 0.40
*Micro.* sp	Ni	Ni	Ni	15.30 ± 0.50
*C. neoformans*	Ni	Ni	Ni	14.30 ± 0.30

Values are mean ± SEM (*n* = 3); ZI: Zone of inhibition (mm); ZI, below 8 mm were considered as less sensitive and were discarded; Ni, No inhibition.

### 3.7 Clot lysis activity

All the selected extracts at 500 µg/100 µL exhibited significant (*p* < 0.05) clot lysis capacity as compared to the control (vehicle) group. Among the extracts, ECT exhibited better clot lysis capacity (61.71 ± 0.03) than the CCT and HCT. However, the standard streptokinase (equiv. 30,000 IU)/100 µL exhibited more clot lysis capacity than the test samples (Table 8).

**TABLE 8:** Clot lysing capacity of the test and controls.

Treatments (100 µL)		Clot lysis (%)
Distilled water (control)		3.72 ± 0.01
Streptokinase (standard) (30,000 IU)		85.56 ± 0.03*
Conc. 500 μg	ECT	61.71 ± 0.03*
	CCT	37.80 ± 0.02*
	HCT	15.19 ± 0.04*

Values are mean ± SEM (*n* = 5); One-way ANOVA, followed by *t*-student *post hoc* test; *p < 0.05 when compared to the control (vehicle) group; ECT: ethanolic fraction of *Cheilanthes tenuifolia*; CCT, chloroform fraction of *Cheilanthes tenuifolia*; HCT, n-hexane fraction of *Cheilanthes tenuifolia*.

## 4 Discussion

Among the natural products, plants are common sources of traditional healing agents worldwide, especially in the poor and less developed countries (Ashmawy et al., 2023; Mia et al., 2023). On the other hand, people in developed countries are now conscious of the adverse events of modern drugs, thus there is a developing enthusiasm for research into and utilization of plant-mediated products or preparations (Abdelazim et al., 2024; Shill et al., 2024). Plant extracts contain numerous groups and components, including alkaloids, glycosides, phenolics and flavonoids, vitamins, and minerals (Gunathilake et al., 2018). Certain groups of compounds have diverse bioactivities, for example, phenolics and flavonoids have promising antioxidant and anti-inflammatory activities (Tlili et al., 2013) and play many important roles in biological systems such as anti-inflammatory (Ginwala et al., 2019), antibacterial, anti-cancer (Khalil et al., 2020), and anti-atherosclerotic effect (Salvamani et al., 2014). A certain study reported that certain organic polar solvents, such as ethanol and methanol, are the best solvents for the extraction of phenolics from natural sources (Ballard et al., 2009). Alkaloids, on the contrary, play vital roles in plants and humans. It is because these are considered defensive compounds. Alkaloids also regulate the growth of plants (Chik et al., 2013). Furthermore, glycosides have many significant therapeutic potentials, including antifungal (Khan et al., 2017) and anticancer (Khan et al., 2019) activities. Current drug development methodologies and modern medicine do not use complete plant extracts and instead rely on specific compounds. Taking the whole plant or extracts without isolating components as practiced in traditional medicine has a stronger therapeutic impact than specific substances (Wen et al., 2005; Thomford et al., 2018). Therefore, the isolated chemical constituents are important for developing novel drugs.

The plant is evidently composed of steroids, alkaloids, flavonoids, triterpenoids, phenolic compounds, and tannins (Ghorpade et al., 2015). Another study reports that the plant is rich in phenolics and flavonoids. It contains quercetin, rutin, and stigmasterol (Mahfuz et al., 2019). Our study also confirmed that the plant contains stigmasterol. In this study, we have seen that ethanolic leaf fractions of C. tenuifolia contain flavonoids. Additionally, we have also found that the plant leaf contains alkaloids, steroids, saponins, glycosides, and reducing sugars. Thus, the compounds of these phytochemical groups may be linked to the observed bioactivities. However, safety pharmacology is critical throughout the drug discovery and development process. Prior to first-in-human investigations, safety pharmacology assays, tests, and models anticipate the clinical risk profile of a possible new medicine (Morimoto et al., 2015). According to the findings of our study the crude ECT up to a 3,000 mg/kg oral dose did not show any behavioral changes or toxicological symptoms, or even death, in Swiss mice. Previous study on the plant extract showed that the LC_50_ values of the chloroform, n-hexane, methanol soluble and ethyl acetate extracts were 34.493 μg/mL, 205.984 μg/mL, 751.169 μg/mL and 66.235 μg/mL respectively on shrimp nauplii (Mahfuz et al., 2019).

Oxidative stress from various sources due to an overabundance of reactive species production (e.g., ROS, RNS) is considered a precursor of diseases and disorders in humans. This can lead to neurological and cardiovascular diseases, cancer, diabetes, etc. (Rekatsina et al., 2020; Forman and Zhang, 2021). Antioxidants are the substances that act against oxidative stress. External antioxidants are required once our body’s defensive mechanisms, including physiological antioxidants, fail to manage an overabundance of free radicals. Plant-based natural antioxidants play significant roles in managing this situation (Jamshidi-Kia et al., 2020). It is due to their being readily available, economic, and biocompatible. Moreover, we can readily access these compounds through our daily diets (Landete, 2013). In a recent experiment, Mahfuz et al. (2019) demonstrated that methanolic whole plant extract exhibited strong (IC_50_ = 9.926 μg/mL) DPPH free radical scavenging capacity. Another investigation of the plant’s methanolic extract found that the two flavonoids (rutin and quercetin) present in the extract showed considerable *in vitro* anti-oxidant activity; in this regard, the DPPH scavenging capability of quercetin (86.1%) was higher than that of rutin (73.2%) (Jarial et al., 2018; Daryono and Rhomawati, 2020; Daryono and Rhomawati, 2020). Another bioactive triterpenoid isolated from the n-hexane extract of the plant also has potent antioxidant capacity such as stigmasterol (Mahfuz et al., 2019), which significantly reduced the ROS generation in different *in vitro* investigation (Agatonovic-Kustrin et al., 2018; Bakrim et al., 2022).

In this investigation, we have seen that all the fractions of C. tenuifolia revealed significant DPPH free radical scavenging capacity in a concentration-dependent manner, with the IC_50_ values estimated for the ECT, HCT, and CCT within the range of 22.17 ± 1.90 to 78.13 ± 2.08 μg/mL, respectively. ECT exhibited a better DPPH radical scavenging effect than the other two fractions. It is because of the large amount of phenolics and flavonoids in this fraction such as rutin and quercetin as well as terpenoids including stigmasterol (Jarial et al., 2018; Mahfuz et al., 2019). The promising DPPH radical scavenging effect of other species of Cheilanthes such as C. anceps also reported by (Chowdhary et al., 2010).

Oxidative stress can provoke inflammatory cascades. Thus, antioxidants have protective effects in a biological system. Certain antioxidants, such as polyphenols, have an anti-ageing effect (Moliner et al., 2020), and these can prevent or delay many disease conditions, including neurological diseases and disorders, cardiovascular diseases, diabetes, and so on (Rodríguez-Yoldi, 2021; Bhuia et al., 2023b). A study performed by Mahfuz and his coworkers in 2019 suggests that the methanol, n-hexane, ethyl acetate, and chloroform fractions of C. tenuifolia showed significant membrane stabilizing capacity in the HRBC assay, where the percent inhibition of hemolysis was determined within the range of 2.97 and 73.97 (Mahfuz et al., 2019). The n-hexane fraction showed the highest percent inhibition against hypnotic solution-induced hemolysis (73.97%), while the ethyl acetate fraction showed the highest percent inhibition of heat-induced hemolysis (67.27%). Our study demonstrated that the organic fractions of C. tenuifolia, especially its ECT, exhibited significant egg protein and HRBC protection capabilities. Another study by Akbor et al. (2023) also stated that methanolic extract of the plant has a notable membrane lysing inhibitory activity (1.42% ± 0.02%) on HRBCs (Akbor et al., 2023). One report suggested that the species C. farinose is traditionally used to manage inflammation (Yonathan et al., 2006). Thus, our present findings are in agreement with the traditional values and scientific reports observed in the databases. According to the findings of different studies the bioactive substances (rutin, and quercetin) of the plant has potent anti-inflammatory activity and liable for diminishing different inflammatory markers such as cytokines (Abd Nikfarjam et al., 2017; Bhuia et al., 2023c).

Infectious diseases due to microbial attacks (e.g., bacteria, fungi, viruses, and parasites) in humans are a common consequence in the world. Among the wide variety of natural products, plants are considered major sources of antimicrobial agents (Touati et al., 2018). The species of the Pteridaceae family are known for their diverse bioactivities, including antioxidant, anti-inflammatory, antimicrobial, anti-cancer, antidiabetic, and neurobiological properties (Baskaran et al., 2018). To date, a number of bioactive compounds have been introduced from the Cheilanthes genus, for example, glycosides of apigenin, chrysoeriol, luteolin, kaempferol, and quercetin from C. concolor, *C. flexuosa* and C. goyazensis (Salatino and Prado, 1998). In this study, we have seen that all the organic fractions of C. tenuifolia acted against both gram (+) and gram (−) species as well as many pathogenic fungi, suggesting its broad-spectrum anti-microbial effects. Conventional antimicrobial agents have some potential adverse effects, for example, allergic reactions and enhanced resistance (Mohsen et al., 2020). For example, chloramphenicol is widely used in meningitis, but it increases the risk of aplastic anemia. Another example is sulfonamides, which were considered “wonder drugs,” but these are evidently contributing to severe skin reactions (King et al., 2018). Therefore, it is crucial to inhibit microbial pathogenesis using appropriate and safe antimicrobials (French, 2005). Previous study by Jarial et al. (2018) also manifested the antimicrobial properties of the plant and the study also revealed that isolated flavonoids such as quercetin and rutin from C. tenuifolia showed inhibitory effects against *Staphylococcus aureus* and *Enterobacter* sp. with MIC values of (2.25 and 0.45 μg/mL), respectively (Jarial et al., 2018).

Atherothrombosis is one of the major causes of morbidity and mortality in the world. Chronic pathology is responsible for vascular remodeling through various pathways, including oxidative stress (Martin-Ventura et al., 2017). ROS and RNS play important pathophysiological roles in vascular diseases, such as atherothrombosis (Chen et al., 2018). It is also evident that microorganism-mediated infections result in the production of potentially reactive molecules in our body through oxygen metabolism (Brown et al., 2014), which is responsible for many pathological conditions, including inflammation, atherosclerosis, carcinogenesis, and so on (Aruoma, 1998). The methanolic whole plant extract is evidently able to exert significant clot lysis capacity at 100 µg/tube (Mahfuz et al., 2019). The authors demonstrated that the ethyl acetate, n-hexane, methanol, and chloroform fractions exhibited 31.59, 12.10, 17.01, and 41.26% clot lysis capacities, respectively. Another study by Akbor et al. (2023) also reported that the methanolic extract of the plant hindered hemolysis in a concentration-dependent manner and inhibited 78.93% ± 0.01% hemolysis (IC_50_ = 46 ± 2.11 μg/mL) at the higher concentration (160 μg/mL) (Akbor et al., 2023). Findings of our study suggest that ECT, CCT, and HCT showed clot lysis capacities of 61.71 ± 0.03, 37.80 ± 0.02, and 15.19% ± 0.04% at 500 µg/tube. ECT showed better clot lysis capacity than the other two fractions. Due to the presence of different phytochemicals such as alkaloids, flavonoids, and steroids (Uddin et al., 2019; Tabassum et al., 2022), the bioactive phytoconstituents of the plant such as rutin, quercetin, and stigmasterol also have remarkable hemolytic activity (Zaragoza et al., 2021; Bari et al., 2022; Chen et al., 2022). We suppose that the observed clot lysis capacity, especially by the ECT, might be linked to the existence of phytochemical groups such as alkaloids, glycosides, and flavonoids and bioactivity such as radical scavenging, anti-inflammatory, and anti-microbial activities. Natural products and their structural counterparts have historically made significant contributions to pharmacology, particularly in cancer and infectious disorders (El-Nashar et al., 2024). Nonetheless, natural products pose hurdles for drug development, such as technical barriers to screening, isolation, characterization, and optimization, which have contributed to a drop in their pursuit by the pharmaceutical industry since the 1990s (Atanasov et al., 2021; El-Nashar et al., 2023a).

## 5 Conclusion and future prospects

As a conclusion, the organic fractions of C. tenuifolia leaf extract remain rich in many valuable phytochemical groups, including alkaloids, tannins, glycosides, flavonoids, and saponins. All the fractions exhibited concentration-dependent and significant radical scavenging, anti-inflammatory, and anti-microbial effects against different gram (+) and gram (−) bacteria, as well as a number of pathogenic fungi, and clot lysis capacities. ECT exhibited better anti-radical (IC_50_ = 22.17 ± 1.90), anti-inflammatory, anti-bacterial, and clot-lysis capacity than the CCT and HCT. We suppose that all the activities, because of the presence of alkaloids, glycosides, and flavonoids within the organic fractions of C. tenuifolia leaf extract and the bioactive compound rutin, quercetin and stigmasterol play an important role in exerting therapeutic activities against oxidative stress and inflammation related diseases and disorders, pathogenic infections and a good source of thrombolytic agents. However, additional research is deemed necessary to conduct the isolation, identification, and clarification of the underlying molecular mechanisms responsible for each individual bioactivity as well as clinical investigation of the plant’s extract and exploited phytochemicals which would be useful in producing plant-based pharmaceuticals to treat numerous complicated human diseases.

## Data Availability

The raw data supporting the conclusion of this article will be made available by the authors, without undue reservation.
